# Expedited Transplant Allocation Using a Paired Kidney Cohort

**DOI:** 10.1001/jamanetworkopen.2026.0257

**Published:** 2026-03-04

**Authors:** Miko E. Yu, S. Ali Husain, Emma G. Tucker, Prateek Sahni, David C. Cron, Jesse D. Schold, Joel T. Adler, Sumit Mohan

**Affiliations:** 1 Vagelos College of Physicians & Surgeons, Columbia University, New York, New York; 2 Columbia University Renal Epidemiology Group, New York, New York; 3 Department of Surgery, Massachusetts General Hospital, Boston; 4 Department of Epidemiology, School of Public Health, University of Colorado–Anschutz Medical Campus, Aurora; 5 Department of Surgery, University of Colorado–Anschutz Medical Campus, Aurora; 6 Department of Surgery and Perioperative Care, Dell Medical School, University of Texas at Austin, Austin; 7 Department of Epidemiology, Mailman School of Public Health, Columbia University, New York, New York

## Abstract

**Importance:**

Organ procurement organizations are increasingly turning to expedited (ie, out-of-sequence) allocation of deceased donor kidneys, citing maximal utility of kidneys at risk of nonuse.

**Objective:**

To identify and compare kidney pairs from the same donor from whom 1 kidney was transplanted in sequence and the other out of sequence.

**Design, Setting, and Participants:**

This retrospective cohort study used US kidney transplant registry data obtained from the Scientific Registry of Transplant Recipients to identify kidney transplant donors from whom at least 1 deceased donor kidney was transplanted out of sequence in 2020 through 2024.

**Main Outcomes and Measures:**

Incidence of unilateral out-of-sequence transplants, in which 1 kidney was placed in sequence and the other kidney from the same donor was placed out of sequence, and survival outcomes of recipients.

**Results:**

A total of 15 602 kidneys from 8544 deceased donors (5304 [62.1%] male; mean [SD] age at organ recovery, 44.4 [15.1] years) with at least 1 kidney transplanted out of sequence were included in the analysis. Of these, 4313 donors (50.5%) had a unilateral kidney placed out of sequence. Compared with recipients of unilateral in-sequence kidneys, recipients of unilateral out-of-sequence kidneys included a greater percentage of older (median [IQR] age at transplant, 60.0 [50.0-67.0] vs 57.0 [47.0-65.0] years), Asian (472 [10.9%] vs 282 [6.5%]), White (2391 [66.1%] vs 2197 [50.9%]), and male (2850 [66.1%] vs 2453 [56.9%]) recipients, as well as those with private insurance (1255 [29.1%] vs 990 [23.0%]) and preemptive transplant (739 [17.1%] vs 438 [10.2%]) (all *P* < .001). Unilateral out-of-sequence kidneys accounted for 38.2% of all out-of-sequence transplants over the course of the study period. From 2020 through 2024, unilateral out-of-sequence kidneys increased from 97 instances from 15 organ procurement organizations to 1698 kidneys from all 56 organizations in the US. The first instance of an out-of-sequence refusal code (ie, the point in the waiting list at which kidneys were first placed out of the standard sequence) decreased from median (IQR) sequence number 393 (155-889) in 2020 to 28 (11-77) in 2024. The proportion of donor-related reasons for refusal decreased 10% to 15% during the study period. In adjusted Cox models, outcomes for unilateral out-of-sequence transplant were not significantly different for patient survival (hazard ratio, 0.84 [95% CI, 0.70-1.02]; *P* = .08) or graft survival (hazard ratio, 0.87 [95% CI, 0.70-1.08]; *P* = .20), compared with unilateral in-sequence transplant.

**Conclusions and Relevance:**

Findings from this cohort study of unilateral out-of-sequence transplants highlight the importance of understanding donor and allocation factors that resulted in bypassing standard allocation and are critical to designing future iterations of the organ allocation system with improved organ use rates.

## Key Points

**Question:**

How frequently does out-of-sequence allocation of a kidney follow in-sequence placement of a kidney from the same donor, and what are the survival outcomes of recipients?

**Findings:**

In this cohort study of 15 602 kidneys from 8544 deceased donors, unilateral out-of-sequence allocation increased 17-fold, with the first attempt occurring at a median sequence number of 28 in 2024 (vs 393 in 2020). There were no significant differences in patient or graft outcomes between unilateral out-of-sequence and unilateral in-sequence transplants.

**Meaning:**

Restricting analyses to cases in which the allocation system was successful for the contralateral kidney raises questions about how organ procurement organizations decide to perform out-of-sequence allocation, given that neither organ quality nor timing appeared to be associated with this decision.

## Teaser

This cohort study estimates the incidence of unilateral out-of-sequence transplants, in which 1 kidney is placed in sequence and the other kidney from the same donor is placed out of sequence.

## Introduction

For patients with end-stage kidney disease, kidney transplantation is the preferred treatment, conferring better survival and quality of life compared with dialysis. However, deceased donor kidneys are a limited resource, meaning approximately 90 000 candidates wait each year for a suitable transplant in the US. In light of this shortage, an objective algorithm based on national allocation policy is used to match deceased donor kidneys to wait-listed candidates and generate a list of patients in order of priority, commonly referred to as the match run. When this match run is generated, eligible candidates are numbered sequentially based on their prioritization, which is primarily based on waiting time but also incorporates factors such as allosensitization, pediatric priority, and proximity measures.^1^

In recent years, regulatory metrics have applied pressure on organ procurement organizations (OPOs), including holding them accountable for transplantation rates for procured organs.^2,3,4,5^ In response to this oversight, as well as added logistical complexities stemming from allocation system revisions in 2021, OPOs are increasingly turning to the out-of-sequence pathway, which had been historically reserved for exigent circumstances.^6,7,8,9,10^

Under standard allocation processes, transplant centers are contacted according to the order of the match run to determine if the center is willing to accept the kidney on behalf of the patient on their waiting list. Out-of-sequence allocation bypasses this order, allowing OPOs to offer the kidney to a transplant center perceived to be more aggressive, that is, more likely to use the organ.^7^ The recipient center can then place the kidney with any of their patients regardless of priority. This process results in passing over transplant centers that are not even aware that there was a kidney available for their patients. Concerningly, this process currently lacks standardization and oversight, and kidneys can be deemed out of sequence at any point in the match run, at the discretion of the OPO.^8,10,11^ The stated rationale for using the out-of-sequence pathway includes increased efficiency in placement, logistical considerations, and maximizing utility given that these are kidneys from less-than-ideal donors and would be otherwise difficult to place, thereby preventing the nonuse of organs.^12^

In this analysis, we identify the incidence and timing of out-of-sequence allocation following an in-sequence placement of the contralateral kidney from the same donor. We examined recipient outcomes and quantified the time taken for OPOs to decide that a kidney would be difficult to place, particularly in cases in which the partner kidney from a donor pair had been successfully placed using the standard allocation process. Examining current practice patterns in out-of-sequence allocation is an important step in understanding why OPOs opt for this pathway and what unintended effects may arise as a result. This analysis is particularly salient given the challenge of accurately capturing donor kidney quality and the widespread interest in developing a standard expedited allocation pathway.

## Methods

We performed a retrospective cohort study using US transplant registry data and potential recipient data. This study used data from the Scientific Registry of Transplant Recipients (SRTR). The SRTR data system includes data on all donors, wait-listed candidates, and transplant recipients in the US, submitted by the members of the Organ Procurement and Transplantation Network (OPTN). The Health Resources and Services Administration (HRSA), US Department of Health and Human Services, provides oversight to the activities of the OPTN and SRTR contractors. The data reported here have been supplied by the Hennepin Healthcare Research Institute (HHRI) as the contractor for the Scientific Registry of Transplant Recipients (SRTR). The interpretation and reporting of these data are the responsibility of the authors and in no way should be seen as an official policy of or interpretation by the SRTR or the US Government. This study used potential transplant recipient match run data available through 2024 and standard analysis files from the SRTR dated March 2025. The Columbia University Irving Medical Center institutional review board approved this study. The requirement for informed consent was waived, as this study used deidentified data from a national transplant registry. All research activities were consistent with the principles of the Declaration of Istanbul^13^ and followed the Strengthening the Reporting of Observational Studies in Epidemiology (STROBE) reporting guideline for cohort studies.

We identified all transplanted kidneys in the US with match runs generated from 2020 through 2024 (n = 92 258). Altruistic nondirected living donor transplants with match runs generated (n = 17 kidneys) were excluded, as well as kidney pairs from a single donor (donor pairs) in which each kidney was allocated on separate match runs (n = 8 kidneys).

Out-of-sequence offers were defined as offers with primary or secondary refusal codes: 861: Operational OPO (n = 271 566 offers), 862: Donor medical urgency (n = 3675 offers), or 863: Offer not made due to expedited placement attempt (n = 13 759 329 offers). Offers with refusal code 799: Other, specify were also identified as out-of-sequence when their accompanying free-text fields contained at least 1 of the following terms: expedited, aggressive, open offer, or out of sequence (n = 1 057 286 offers).^10^ Organ offers that followed the standard allocation policy are referred to as in-sequence.

Kidneys were considered transplanted out of sequence when the out-of-sequence refusal code appeared earlier in the match run than the eventual organ acceptance code. For example, consider a pair of kidneys from the same donor that were transplanted at sequence number 30 and 150, respectively. If the first appearance of an out-of-sequence code occurred at sequence number 100, then the first transplanted kidney was in sequence, whereas the second transplanted kidney was out of sequence.

We excluded donors with no kidneys transplanted out of sequence (n = 76 631 kidneys) and grouped our final study cohort of 15 602 kidneys as follows: (1) donor pairs with both kidneys transplanted out of sequence (bilateral out of sequence), (2) donors pairs with 1 kidney transplanted in sequence (unilateral in sequence) and 1 kidney transplanted out of sequence (unilateral out of sequence), and (3) single kidney out-of-sequence transplants.

For all out-of-sequence transplants, we compared the sequence numbers of the first appearance of an out-of-sequence code at the organ level and at the center level. We counted the number of transplant centers that received offers on the match run prior to the first out-of-sequence refusal code. For each year, we also assessed the distribution of the OPOs’ median first out-of-sequence appearance. Organ travel distance between the OPO and transplant center was obtained by using the latitudes and longitudes of OPOs and transplant centers and computing their geodetic distances in nautical miles.

For all donor pairs with a unilateral transplant, we compared the placement sequence of the in-sequence kidney vs the out-of-sequence kidney. We further stratified the kidneys by whether they were transplanted at the same center or not and compared the absolute difference in placement sequence number. We also examined the distribution of all reasons for refusal entered by transplant centers prior to the first out-of-sequence code by classifying refusal reasons into 7 categories: recipient, donor history, biopsy findings, organ damage or anatomy, logistic, cold ischemia time, or other (eTable in Supplement 1).

We compared outcomes for kidneys from donors in which 1 kidney was placed in-sequence and the other out-of-sequence. Recipient demographics (age at transplant, sex, race, ethnicity, educational level, employment status, primary payor) and clinical characteristics (history of diabetes, cold ischemia time, kidney donor profile index [KDPI], preemptive transplant status, and time receiving dialysis) were summarized within each of the groupings. Race and ethnicity variables used were center-reported social variables that were classified by the SRTR and included Asian, Black, Hispanic, White, and other, which comprised multiracial, Native American, and Pacific Islander. Race and ethnicity were assessed to characterize the study cohort. KDPI, an indicator of kidney quality that ranges from 0% to 100%, with higher percentages indicating shorter estimated function and is used as part of the allocation algorithm, was calculated using the scaling factor of each respective year under the race- and hepatitis C virus status-inclusive equation since the KDPI refit equation was first implemented in October 2024.

### Statistical Analysis

Comparisons of recipients within the same donor pair were assessed using the Wilcoxon signed-ranked test for continuous variables; the McNemar test was used for binary variables, and the Bowker test of symmetry was used for categorical variables with more than 2 categories. We also compared outcomes for out-of-sequence kidneys between unilateral, bilateral, and single kidney donors using pairwise McNemar-Bowker and Kruskal-Wallis tests. To compare patient survival after transplant and death-censored graft survival for recipients of kidneys from unilateral donor pairs, we estimated outcomes using Cox proportional hazards models with robust sandwich estimators to account for paired donation. Cox models were also adjusted for recipient characteristics (age, race, gender, educational and employment status, history of diabetes, and insurance type). Cox models were also stratified by KDPI (<60% and ≥60%) to examine the associations of donor characteristics with patient and graft survival.

All tests were 2-sided. Statistical significance was determined as the 95% CI excluding 1, and analyses were performed using STATA/MP 18.0 (StataCorp LLC).

## Results

**Figure 1.**
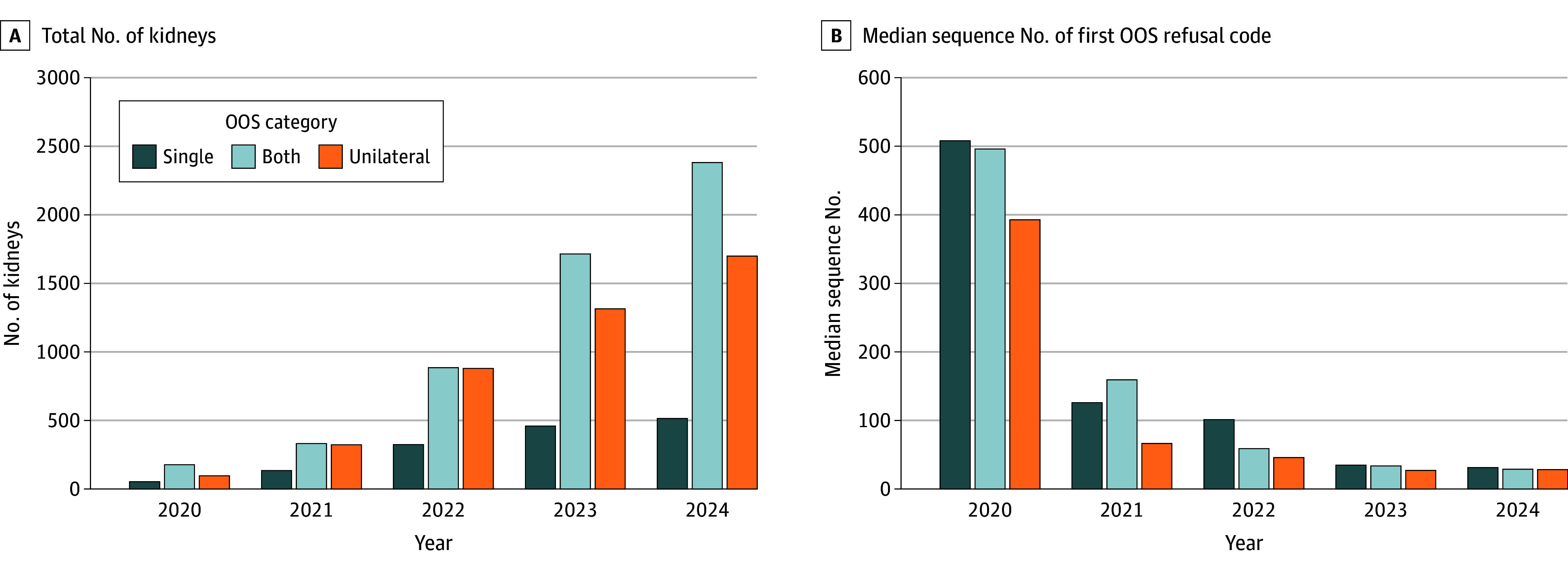
Bar Graph Depicting Annual Number of Kidneys Transplanted and Median Sequence Number of First Appearance of Out-of-Sequence (OOS) Transplant Refusal Code in 3 Cohorts Both indicates donor pairs with both kidneys transplanted OOS; unilateral, 1 kidney transplanted OOS while its contralateral kidney was transplanted in-sequence; single, single kidney transplanted OOS.

A total of 15 602 kidneys from 8544 donors 3240 [37.9%] female and 5304 [62.1%] male; mean [SD] age at organ recovery, 44.4 [15.1] years) with at least 1 kidney transplanted out of sequence were included in the analysis (eFigure 1 in Supplement 1). Donors were 180 (2.1%) Asian, 1311 (15.4%) Black, 6897 (80.7%) White, and 156 (1.8%) other race, and 1132 (13.9%) were Hispanic ethnicity. Of 8544 donors, 2745 (32.1%) had both kidneys transplanted out of sequence (n = 5490 kidneys), 1486 (17.4%) had only a single kidney procured and placed out of sequence, and 4313 (50.5%) had a unilateral kidney placed out of sequence with the other kidney placed in sequence (n = 8626 kidneys). Overall, unilateral out-of-sequence kidneys accounted for 38.2% of all out-of-sequence kidney transplants, and this category experienced the fastest growth during the study period, increasing 17-fold, whereas single and bilateral out-of-sequence transplants increased approximately 10- and 13-fold, respectively (Figure 1). By the end of the study period, 4595 out-of-sequence kidney transplants occurred in 2024, accounting for nearly 1 in 4 (22.6%) of all deceased donor transplants that year (Table 1).

**Table 1.** Annual Patterns of OOS Transplants, 2020-2024

Characteristic	Year				
	2020	2021	2022	2023	2024
**Donor pairs: bilateral OOS**					
Total No. of OOS kidneys	178	332	884	1714	2382
KDPI, median (IQR)^a^	54 (36-73)	51 (28-68)	57 (36-76)	57 (38-74)	64 (46-80)
Sequence No. of the first OOS refusal code, median (IQR)	496 (175-905)	159 (33-497)	59 (14-298)	34 (9-136)	29 (10-85)
No. of centers offered on the match run prior to the first OOS refusal code, median (IQR)	12 (10-17)	16 (7-29)	12 (6-25)	10 (4-19)	10 (5-16)
Kidney pairs transplanted at the same center, %	87.6	65.5	60.0	49.6	44.5
**Donor pairs: unilateral OOS**					
Total No. of OOS kidneys	97	323	881	1315	1698
KDPI, median (IQR)^a^	54 (35-77)	51 (26-73)	48 (26-72)	48 (29-66)	55 (35-74)
Sequence No. of the first OOS refusal code, median (IQR)	393 (155-889)	66 (19-269)	46 (15-200)	27 (12-90)	28 (11-77)
No. of centers offered on the match run prior to the first OOS refusal code, median (IQR)	14 (10-21)	13 (8-25)	13 (7-22)	10 (6-17)	10 (6-16)
Kidney pairs transplanted at the same center, %	7.2	15.2	13.9	14.2	14.8
No. of OPOs that performed a unilateral OOS offer	15	46	56	55	56
**Single kidney OOS**					
Total No. of OOS kidneys	53	134	324	460	515
KDPI, median (IQR)^a^	71 (41-80)	67 (29-92)	64 (32-83)	58 (34-78)	63 (37-80)
Sequence No. of the first OOS refusal code, median (IQR)	508 (57-972)	126 (25-441)	101 (15-427)	35 (8-203)	31 (8-175)
No. of centers offered on the match run prior to the first OOS refusal code, median (IQR)	12 (7-19)	13 (6-30)	14 (5-26)	9 (4-19)	9 (4-15)

Abbreviations: KDPI, Kidney Donor Profile Index; OOS, out of sequence; OPO, organ procurement organization.

^a^ KDPI ranges from 0% to 100%, with higher values indicating shorter estimated function.

During the study period, the overall median (IQR) sequence number for the first appearance of an out-of-sequence code among all out-of-sequence transplants was 37 (12-156), with a rapid decrease from a median (IQR) of 466 (128-905) in 2020 to 29 (11-88) in 2024 (eFigure 2 in Supplement 1). The pattern for an earlier switch to out-of-sequence allocation occurred across all categories of out-of-sequence transplants, that is, single, unilateral, and bilateral (Figure 1).

**Figure 2.**
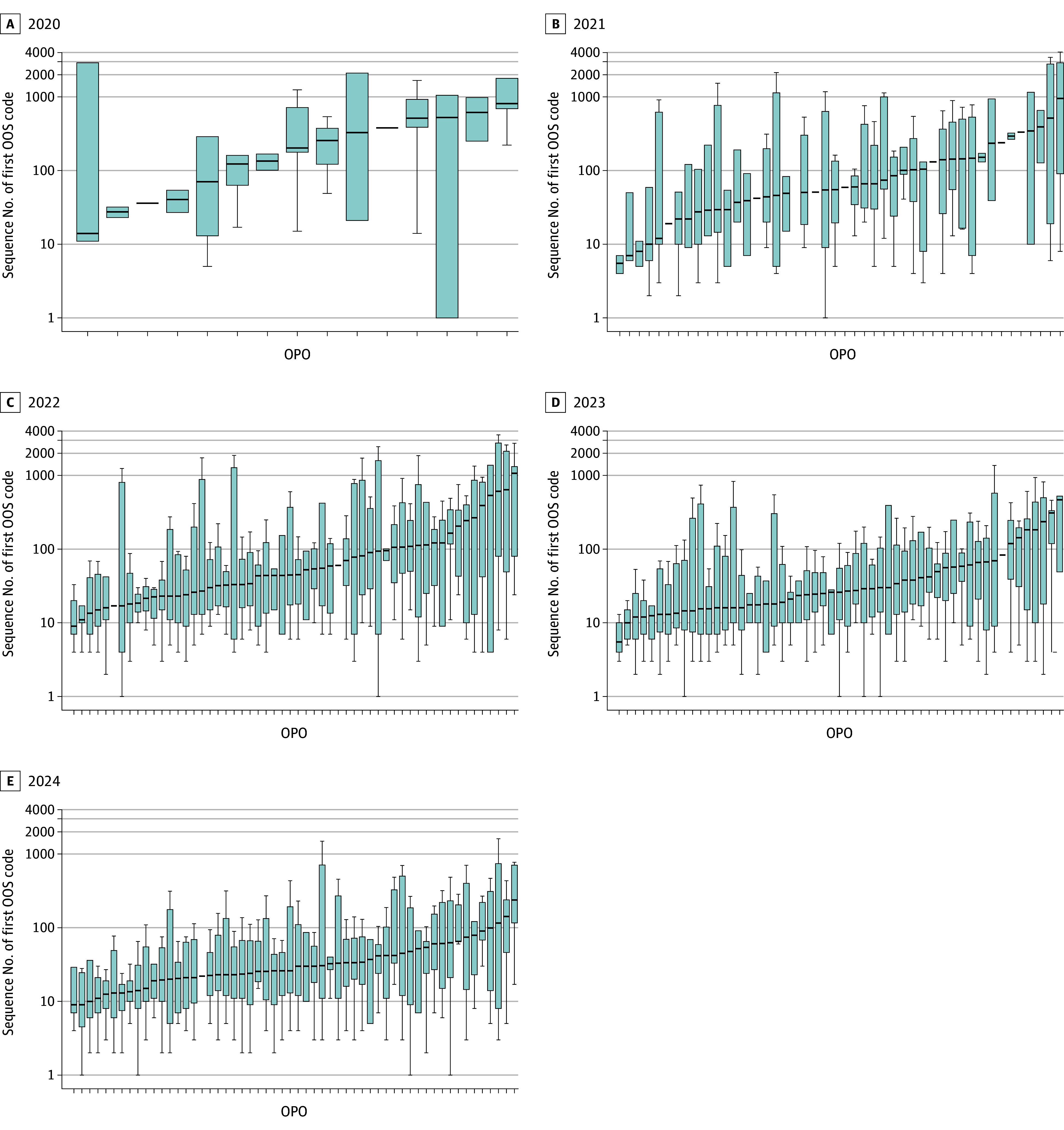
Boxplots Showing the Distribution of the First Appearance of an Out-of-Sequence (OOS) Refusal Code Among Donor Pairs With Unilateral Transplants by Year From 2020 to 2024 Each boxplot represents an organ procurement organization (OPO), showing the number of OPOs with a unilateral kidney (1 kidney transplanted OOS) increasing over time. Boxes represent the IQR, and whiskers extend to values 1.5 times the IQR. Black markers indicate the median sequence number of each OPO.

Among unilateral out-of-sequence transplants performed in 2020, a median (IQR) of 14 (10-21) transplant centers received offers prior to the appearance of the first out-of-sequence refusal code (Table 1). This remained constant until 2023, when it decreased to a median (IQR) of 10 (6-17) transplant centers and remained constant in 2024. The proportion of unilateral out-of-sequence kidneys that went to the same center that accepted the first kidney from the same donor in sequence doubled from 7.2% (n = 7) to 14.8% (n = 251) during the study period. From 2020 to 2024, the number of unilateral out-of-sequence kidneys increased from 97 instances across 15 OPOs to 1698 kidneys across all 56 OPOs. The KDPI of these kidneys ranged from 1% to 100%, whereas the median KDPI stayed relatively constant. The median (IQR) sequence number when OPOs started the out-of-sequence allocation after a successful in-sequence allocation decreased from 393 (155-889) in 2020 to 28 (11-77) in 2024 (Figure 2).

Unilateral out-of-sequence kidneys that were placed out of sequence earlier in the match run were compared with kidneys allocated out of sequence later (eFigure 3 in Supplement 1). Those allocated out of sequence before sequence 100, which do not meet the commonly used definition for a hard-to-place kidney, had a higher proportion of recipient factors cited as reasons for decline, such as a positive crossmatch or a candidate medically unsuitable, and a 10% to 15% lower proportion of donor-related factors, such as organ damage or anatomy, biopsy findings, or donor history (eFigure 4 in Supplement 1). Additionally, there were changes in the prevalence of different refusal codes over time. For example, in 2020, the overwhelming majority of refusal codes, regardless of the sequence number at which the first out-of-sequence code was reported, were related to donor history, which was the single largest category followed by organ damage or other. In contrast, by 2024, donor history represented a minority of instances, and other was the single largest category.

Recipients of unilateral out-of-sequence kidneys (n = 4313) included a greater proportion of older (median [IQR] age at transplant, 60.0 [50.0-67.0] vs 57.0 [47.0-65.0] years), Asian (472 [10.9%] vs 282 [6.5%]), White (2391 [55.4%] vs 2197 [50.9%]), and male (2850 [66.1%] vs 2453 [56.9%]) recipients, as well as recipients with college degrees or higher (1289 [29.9%] vs 1100 [25.5%]), recipients with private insurance (1225 [29.1%] vs 990 [23.0%]), and recipients with preemptive transplant (739 [17.1%] vs 438 [10.2%]), compared with recipients of unilateral in-sequence kidneys (n = 4313) (all *P* < .001) (Table 2). Compared with recipients of bilateral out-of-sequence kidneys (n = 5490), unilateral out-of-sequence recipients (n = 4313) were younger (mean [IQR] age at transplant, 60.0 [50.0-67.0] vs 62.0 [54.0-68.0] years), with a lower percentage of diabetes (1922 [44.6%] vs 2691 [49.0%]) and preemptive transplant (739 [17.1%] vs 1115 [20.3%]) and higher median (IQR) sequence numbers at transplant (462 [108-1808] vs 82 [227-2532]) (all *P* < .001).

**Table 2.** Recipient Characteristics of the Study Cohorts, 2020-2024

Recipient characteristic	Donor pair			Single kidney: OOS (n = 1486)	*P* value			
	Bilateral OOS (n = 5490)	Unilateral OOS (n = 4313)	Unilateral InS (n = 4313)		Unilateral OOS vs unilateral InS	Unilateral OOS vs bilateral OOS	Unilateral OOS vs single OOS	Bilateral OOS vs single OOS
Age at transplant, median (IQR), y	62.0 (54.0-68.0)	60.0 (50.0-67.0)	57.0 (47.0-65.0)	61.0 (51.0-68.0)	<.001	<.001	.002	<.001
Sex, No. (%)								
Female	1843 (33.6)	1463 (33.9)	1860 (43.1)	553 (37.2)	<.001	<.001	<.001	<.001
Male	3647 (66.4)	2850 (66.1)	2453 (56.9)	933 (62.8)				
Race, No. (%)								
Asian	552 (10.1)	472 (10.9)	282 (6.5)	184 (12.4)	<.001	.59	.12	.05
Black	1718 (31.3)	1396 (32.4)	1736 (40.3)	456 (30.7)				
White	3114 (56.7)	2391 (55.4)	2197 (50.9)	816 (54.9)				
Other or missing^a^	106 (1.9)	54 (1.3)	98 (2.3)	30 (2.0)				
Ethnicity, No. (%)								
Hispanic or Latino	959 (17.5)	804 (18.6)	835 (19.4)	295 (19.9)	.36	<.001	<.001	<.001
Non-Hispanic	4531 (82.5)	3509 (81.4)	3478 (80.6)	1191 (80.1)				
History of diabetes, No. (%)	2691 (49.0)	1922 (44.6)	1797 (41.7)	659 (44.3)	.005	<.001	<.001	<.001
Educational status, No. (%)								
None or unknown	142 (2.6)	124 (2.9)	152 (3.5)	33 (2.2)	<.001	.006	.19	.56
High school, GED, or less	2202 (40.1)	1813 (42.0)	1949 (45.2)	621 (41.8)				
Some college or technical school	1367 (24.9)	1087 (25.2)	1112 (25.8)	356 (24.0)				
Associate or bachelor’s degree	1206 (22.0)	878 (20.4)	796 (18.5)	328 (22.1)				
Graduate degree	573 (10.4)	411 (9.5)	304 (7.0)	148 (10.0)				
Employment status, No. (%)								
Unemployed	3518 (64.1)	2668 (61.9)	2792 (64.7)	951 (64.0)	<.001	.06	.04	.45
Employed	1601 (29.2)	1348 (31.3)	1145 (26.5)	456 (30.7)				
Unknown	371 (6.8)	297 (6.9)	376 (8.7)	79 (5.3)				
Primary payer at transplant, No. (%)								
Public, other, or unknown	3924 (71.5)	3058 (70.9)	3323 (77.0)	1039 (69.9)	<.001	.55	.35	.60
Private	1566 (28.5)	1255 (29.1)	990 (23.0)	447 (30.1)				
Delayed graft function, No. (%)	2160 (39.3)	1482 (34.4)	1681 (39.0)	514 (34.6)	<.001	<.001	<.001	<.001
Cold ischemia time, median (IQR), h	23.1 (19.4-28.2)	24.1 (20.2-28.9)	19.4 (15.9-23.0)	24.2 (19.8-29.2)	<.001	<.001	.49	<.001
KDPI %, No. (%)^b^								
0-20	352 (6.4)	493 (11.4)	493 (11.4)	192 (12.9)	NA	<.001	<.001	.63
21-40	1002 (18.3)	1001 (23.2)	1001 (23.2)	205 (13.8)				
41-60	1270 (23.1)	1113 (25.8)	1113 (25.8)	286 (19.2)				
61-80	1704 (31.0)	1110 (25.7)	1110 (25.7)	414 (27.9)				
80-100	1162 (21.2)	596 (13.8)	596 (13.8)	389 (26.2)				
Laterality, No. (%)								
Left kidney	2745 (50.0)	1825 (42.3)	2488 (57.7)	416 (28.0)	NA	<.001	<.001	<.001
Right kidney	2745 (50.0)	2488 (57.7)	1825 (42.3)	598 (40.2)				
En bloc	NA	NA	NA	472 (31.8)				
Preemptive transplant, No. (%)	1115 (20.3)	739 (17.1)	438 (10.2)	260 (17.5)	<.001	<.001	<.001	.02
Time receiving dialysis, if not preemptive transplant, median (IQR), y	2.3 (1.3-3.7)	2.7 (1.4-4.4)	4.7 (2.5-7.5)	2.3 (1.2-3.6)	<.001	<.001	<.001	.23
Distance organ traveled from OPO to transplant center, median (IQR), nautical miles	168.1 (25.7-555.2)	163.7 (37.1-340.9)	121.8 (17.1-209.0)	209.5 (70.7-723.8)	<.001	.002	<.001	<.001
Sequence number at acceptance, median (IQR)	82 (227-2532)	462 (108-1808)	9(3-29)	978 (243-3492)	<.001	<.001	<.001	<.001

Abbreviations: GED, General Educational Development; InS, in sequence; KDPI, Kidney Donor Profile Index; NA, not applicable; OOS, out of sequence; OPO, organ procurement organization.

^a^ Other category includes multiracial, Native American, and Pacific Islander.

^b^ KDPI ranges from 0% to 100%, with higher values shorter estimated function.

Recipients of unilateral out-of-sequence kidneys at the same transplant center as their contralateral kidney from the same donor (n = 616), compared with recipients of unilateral out-of-sequence kidneys at a different center (n = 3697), had a lower percentage of recipients with preemptive transplant (88 [14.3%] vs 651 [17.6%]; *P* = .04). Recipients at the same transplant center had more dialysis time (median [IQR], 2.9 [1.6-4.6] vs 2.7 [1.4-4.4] years; *P* = .03) and were transplanted at a lower median (IQR) sequence number (177 [51-493] vs 576 [127-2100]; *P* < .001) (Table 3). Kidneys that went out of sequence to a different center from the one that accepted the in-sequence mate kidney included a larger share of kidneys with a KDPI of 20% or lower and that traveled nearly twice as far (median [IQR], 171.0 [49.4-416.5] vs 98.8 [7.0-199.2] nautical miles) before being transplanted much lower on the match run (median [IQR] sequence number, 576 [127-2100] vs 177 [51-493]).

**Table 3.** Recipient Characteristics of Unilateral OOS Kidneys Allocated to the Same Transplant Center as the Unilateral In-Sequence Kidney Pair, 2020-2024

Recipient characteristic	Unilateral OOS		*P* value
	Different transplant center (n = 3697)	Same transplant center (n = 616)	
Age at transplant, median (IQR),y	60.0 (50.0-67.0)	60.0 (50.0-67.0)	.59
Sex, No. (%)			
Female	1244 (33.6)	219 (35.6)	.36
Male	2453 (66.4)	397 (64.4)	
Race, No. (%)			
Asian	403 (10.9)	69 (11.2)	.87
Black	1191 (32.2)	205 (33.3)	
White	2058 (55.7)	333 (54.1)	
Other or missing^a^	45 (1.2)	9 (1.5)	
Ethnicity, No. (%)			
Hispanic or Latino	697 (18.9)	107 (17.4)	.38
Non-Hispanic	3000 (81.1)	509 (82.6)	
History of diabetes, No. (%)	1650 (44.6)	272 (44.2)	.83
Educational status, No. (%)			
None or unknown	108 (2.9)	16 (2.6)	.98
High school, GED or less	1550 (41.9)	263 (42.7)	
Some college or Technical school	934 (25.3)	153 (24.8)	
Associate or bachelor’s degree	755 (20.4)	123 (20.0)	
Graduate degree	350 (9.5)	61 (9.9)	
Employment status, No. (%)			
Unemployed	2293 (62.0)	375 (60.9)	.06
Employed	1163 (31.5)	185 (30.0)	
Unknown	241 (6.5)	56 (9.1)	
Primary payer at transplant, No. (%)			
Public, other, or unknown	2612 (70.7)	446 (72.4)	.57
Private	1085 (29.3)	170 (27.6)	
Delayed graft function, No. (%)	1242 (33.6)	240 (39.0)	.009
Cold ischemia time, median (IQR), h	24.1 (20.3-29.0)	23.7 (20.2-28.2)	.13
KDPI %, No. (%)^b^			
0-20	451 (12.2)	42 (6.8)	<.001
21-40	869 (23.5)	132 (21.4)	
41-60	954 (25.8)	159 (25.8)	
61-80	939 (25.4)	171 (27.8)	
80-100	484 (13.1)	112 (18.2)	
Laterality			
Left kidney	1567 (42.4)	259 (42.0)	.87
Right kidney	2130 (57.6)	357 (58.0)	
Preemptive transplant, No. (%)	651 (17.6)	88 (14.3)	.04
Time receiving dialysis, if not preemptive transplant, median (IQR), y	2.7 (1.4-4.4)	2.9 (1.6-4.6)	.03
Distance organ traveled from OPO to transplant center, median (IQR), nautical miles^c^	171.0 (49.4-416.5)	98.8 (7.0-199.2)	<.001
Sequence No. at acceptance, median (IQR)	576 (127-2100)	177 (51-493)	<.001

Abbreviations: GED, General Educational Development; KDPI, Kidney Donor Profile Index; OOS, out of sequence; OPO, organ procurement organization.

^a^ Other category includes multiracial, Native American, and Pacific Islander.

^b^ KDPI ranges from 0% to 100%, with higher values indicating shorter estimated function.

In unadjusted Cox models, outcomes for unilateral out-of-sequence transplant were not significantly different in terms of patient survival (hazard ratio [HR], 0.89 [95% CI, 0.75-1.08]; *P* = .24) or death-censored graft survival (HR, 0.86 [95% CI, 0.69-1.06]; *P* = .16) compared with unilateral in-sequence transplant (eFigure 5 in Supplement 1). Similarly, there remained no significant difference after adjusting for recipient characteristics in patient survival (adjusted HR, 0.84 [95% CI, 0.70-1.02]; *P* = .08) or graft survival (adjusted HR, 0.87 [95% CI, 0.70-1.08]; *P* = .20). There were no significant differences between unilateral out of sequence compared with unilateral in sequence when stratifying by KDPI groups (eFigure 6 in Supplement 1).

## Discussion

Our analysis demonstrated a rapid increase in the number of out-of-sequence kidney transplants over time, with 4595 out-of-sequence kidney transplants occurring in 2024, accounting for nearly 1 in 4 (22.6%) of all deceased donor transplants that year. More than a third of these out-of-sequence transplants in 2024 were unilateral out-of-sequence allocations, meaning that they followed the successful placement of the mate kidney in sequence. We also noted that these practices have become more widespread, with OPOs in 2024 allocating these kidneys out of sequence, while doing so much earlier in the match run process. Understanding the factors that are driving this behavior and whether this practice is truly a reflection of donor quality and concerns of nonuse is critical to determining next steps in the development of an expedited pathway for kidneys at risk of nonuse. Given the criticisms of the currently available measures of donor quality, such as KDPI, as not being adequate or complete indicators of kidney quality,^14^ we restricted our analyses to donor pairs with unilateral out-of-sequence transplants, taking into account that both kidneys would be expected to have similar donor-related attributes, barring those donor factors such as anatomical abnormalities or injury that may be unilateral only.

While the volume of out-of-sequence transplants has increased rapidly, this has been particularly stark for unilateral out-of-sequence transplants. The rapid increase in this practice is hard to understand and counters the argument that out-of-sequence placements are necessarily due to organ quality concerns, given that a kidney from the same donor was successfully placed. Additionally, the rapid decrease in the sequence at which OPOs start out-of-sequence allocation, of kidneys across the KDPI spectrum, suggests an increasing level of comfort by the OPO community to use this pathway for organ placement despite the potential adverse consequences for patients who are skipped.^8,15,16,17,18,19^ Our results indicate that the increasing use of the out-of-sequence pathway is associated with inequalities in transplant outcomes and care, with recipients of unilateral out-of-sequence transplants having disproportionately higher rates of educational attainment, private insurance, and preemptive transplant than recipients of their unilateral in-sequence counterparts.^7,11^ Our results also suggest that there are inequalities among recipients of unilateral out-of-sequence transplants that are performed at the same center as its mate kidney, compared with recipients of unilateral out-of-sequence transplants performed at different centers, although the underlying reasons are still unclear.

Arguments supporting out-of-sequence allocation as a workaround for more efficient placement of less-than-ideal kidneys within a complex allocation system may be overstated, given that unilateral in-sequence kidneys were able to be placed successfully early in the match run. Additionally, only a small minority of the unilateral out-of-sequence kidneys were allocated to the center that took the first kidney, which appears counterintuitive if the concern is to avoid failure to find a transplant center willing to use organs from a given donor. Considering that many of the reasons cited for out-of-sequence placement of kidneys are centered around the reluctance of more conservative centers to accept less-than-ideal organs,^20,21^ as well as the logistical and efficiency challenges introduced by KAS250 (Kidney Allocation System 250),^9,17,22^ we would expect OPOs to offer the second kidney in a donor pair to the transplant center that accepted the first kidney. However, our data do not support this rationale. In fact, the majority of unilateral out-of-sequence kidneys were allocated to different centers, resulting in greater accrued cold ischemia time and greater distance traveled by the organ, contradicting the notion of allocation efficiency. This practice raises questions about how an OPO identifies a center that should receive a given unilateral out-of-sequence offer.

Refusal codes entered for this subset of kidneys also call into question the accuracy of reasons for refusal provided by the center, given the large proportion of donor-related refusals among pairs that had 1 kidney transplanted successfully via standard allocation. Conversely, centers that accept 1 less-than-ideal kidney may be reluctant to double down on the risk by accepting both kidneys if the reasons for declining are related to donor factors, necessitating that the OPO find a different center willing to accept the second kidney.

These patterns are of particular concern due to the current lack of consensus and guidance about when the use of this alternative pathway would be justified, the lack of transparency in the process, and the direct implications to patient outcomes. The ability of centers to know that they would be informed when there is an organ available for one of their patients is central to a well-functioning allocation process. Particularly given the introduction of default organ offer filters that are reflective of a center’s prior organ offer acceptance practice, the rationale for skipping over centers seems unclear at best.^15^ The greater flexibility that these offers create for centers to pick the preferred recipient does not improve utility as measured by graft outcomes. This is consistent with prior data that demonstrated that the sequence number at which a kidney is placed within the allocation system is not reflective of the outcome posttransplant.^20^

### Limitations

There are limitations to this study. The practice of ranked open offers requires transplant centers to enter decline codes (instead of bypass codes) up to the sequence number at which the offer was placed. Therefore, it is possible that out-of-sequence transplants were undercounted; however, there is currently no available method of distinguishing these offers in the dataset. The national dataset also does not have information on anatomical injuries or abnormalities that may affect an individual organ, and we acknowledge that a paired kidney design does not account for these factors that can affect just 1 kidney from a donor.

## Conclusions

In conclusion, the results of this cohort study highlight an increase in unilateral out-of-sequence kidney transplants that appeared to be outpacing the rapid growth in out-of-sequence kidney transplantation. By removing concerns about measures of donor quality, these unilateral out-of-sequence transplants raise important questions about the benefits of out-of-sequence allocation given that they are from donors who the allocation system worked for and that their use did not come with improved logistics or outcomes. Our analysis also demonstrated an earlier and increasing use of the out-of-sequence pathway, suggesting increasing comfort with this pathway in the system as well as a willingness to transport these kidneys further, despite the presence of a closer center that was demonstrably willing to accept organs from these donors. These practices are challenging to fully understand with the existing data and practices, underscoring the need for more information and greater transparency in any future expedited organ allocation pathway.

## Notes

Supplement 1. **eTable.** Categorization of refusal codes **eFigure 1.** Flow diagram of study cohort **eFigure 2.** Distribution of the first appearance of an out-of-sequence refusal code (on a logarithmic scale) among all out-of-sequence transplants **eFigure 3.** Annual proportion of refusal codes used before the first appearance of an out-of-sequence code among donor pairs with unilateral transplants **eFigure 4.** Proportion of refusal codes used before the first appearance of an out-of-sequence code among donor pairs with unilateral transplants **eFigure 5.** Unadjusted and adjusted patient and graft survival after transplant among donor kidney pairs with unilateral out-of-sequence transplants **eFigure 6.** Unadjusted and adjusted patient and graft survival after transplant among donor kidney pairs with unilateral out-of-sequence transplants, stratified by Kidney Donor Profile Index (KDPI)

Supplement 2. Data Sharing Statement
