# Supplementary material for: Expedited Transplant Allocation Using a Paired Kidney Cohort
Source: JAMA Netw Open. 2026 Mar 4;9(3):e260257. doi: 10.1001/jamanetworkopen.2026.0257 (PMC12961519; doi:10.1001/jamanetworkopen.2026.0257)
Supplement: Supplement 1. — eTable. Categorization of refusal codes eFigure 1. Flow diagram of study cohort eFigure 2. Distribution of the first appearance of an out-of-sequence refusal code (on a logarithmic scale) among all out-of-sequence transplants eFigure 3. Annual proportion of refusal codes used before the first appearance of an out-of-sequence code among donor pairs with unilateral transplants eFigure 4. Proportion of refusal codes used before the first appearance of an out-of-sequence code among donor pairs with unilateral transplants eFigure 5. Unadjusted and adjusted patient and graft survival after transplant among donor kidney pairs with unilateral out-of-sequence transplants eFigure 6. Unadjusted and adjusted patient and graft survival after transplant among donor kidney pairs with unilateral out-of-sequence transplants, stratified by Kidney Donor Profile Index (KDPI) [file jamanetwopen-e260257-s001.pdf]

Supplementary Online Content

Yu ME, Husain SA, Tucker EG, et al. Expedited transplant allocation using a paired kidney cohort. 2026;9(3):e260257. doi:10.1001/jamanetworkopen.2026.0257

eTable. Categorization of refusal codes

eFigure 1. Flow diagram of study cohort

eFigure 2. Distribution of the first appearance of an out-of-sequence refusal code (on a logarithmic scale) among all out-of-sequence transplants

eFigure 3. Annual proportion of refusal codes used before the first appearance of an out-of-sequence code among donor pairs with unilateral transplants

eFigure 4. Proportion of refusal codes used before the first appearance of an out-of-sequence code among donor pairs with unilateral transplants

eFigure 5. Unadjusted and adjusted patient and graft survival after transplant among donor kidney pairs with unilateral out-of-sequence transplants

eFigure 6. Unadjusted and adjusted patient and graft survival after transplant among donor kidney pairs with unilateral out-of-sequence transplants, stratified by Kidney Donor Profile Index (KDPI)

This supplementary material has been provided by the authors to give readers additional information about their work.

© 2026 Yu ME et al. *JAMA Network Open*.

eTable. Categorization of refusal codes

Category	Refusal code	Description
Recipient	720	Candidate temporarily medically unsuitable
	721	Candidate transplanted or pending transplant
	722	Candidate's condition improved, transplant not needed
	723	Candidate requires different laterality
	724	Candidate requires multi-organ transplant
	725	Epidemic/pandemic - candidate
	726	Candidate temporarily ineligible due to insurance or financial issue
	727	Candidate unavailable
	728	Candidate refused
	730	No candidate serum for crossmatching
	732	Positive physical crossmatch
	733	Positive virtual crossmatch/unacceptable antigens
	734	Number of HLA mismatches unacceptable
	800	Patient's condition improved, transplant not needed
	801	Patient ill, unavailable, refused, or temporarily unsuitable
	802	Multiple organ transplant or different laterality is required
	803	Patient transplanted
	810	Positive crossmatch
	811	Number of HLA mismatches unacceptable
	812	No serum
	813	Unacceptable antigens
	815	High cPRA
	832	Donor ABO
	840	COVID-19; candidate-related reason
	885	Dual kidneys not available - bypass dual kidney potential recipient
Logistic	760	Resource time constraint (OPO, TXC, donor hospital)
	762	Recovery team availability
	763	Transplant team or transplant facility availability
	764	Transportation availability
	790	Disaster emergency management consideration
	820	Heavy workload
	823	Surgeon unavailable
	824	Distance to travel or ship
	825	Operational - transplant center
	842	COVID-19; OPO or transplant hospital operational issue
	861	Operational - OPO
Cold ischemia time	712	Actual or projected cold ischemic time too long
	2001	Cold ischemic time

© 2026 Yu ME et al. *JAMA Network Open*.

Category	Refusal code	Description
Organ damage or anatomy	701	Organ size, specify
	711	Organ anatomical damage or defect
	836	Organ anatomical damage or defect
Biopsy findings	715	Biopsy results unacceptable
Donor history	700	Donor age
	740	PHS risk criteria or social history
	741	Positive infectious disease screening test
	742	Donor infection or positive culture
	743	Malignancy or suspected malignancy
	750	Donor medical history, specify
	751	Donor instability/high vasopressor usage
	752	Prolonged downtime/CPR
	830	Donor age or quality
	831	Donor size/weight
	833	Donor social history
	834	Positive serological tests
	837	Organ-specific donor issue
	883	Not offered - minimum acceptance criteria not met
Other	710	Organ preservation: unacceptable method or findings
	713	Warm ischemic time too long
	714	Biopsy not available
	716	Organ specific test results not available, specify
	717	Unacceptable organ specific rest results, specify
	731	No donor cells/specimen for crossmatching
	744	Epidemic/pandemic - donor
	753	DCD donor neurological function/not expected to arrest
	761	Donor family time constraint
	765	Exceeded policy response time
	798	Other, specify
	799	Other, specify
	822	Exceeded one hour response time
	835	Organ preservation
	841	COVID-19; donor-related reason
	850	Multi-organ placement
	851	Directed donation
	852	Military donor
	853	ALU, sharing agreement, variance
	860	Medical urgency of another potential recipient
	862	Donor medical urgency

© 2026 Yu ME et al. *JAMA Network Open*.

Category	Refusal code	Description
	880	Kidney placed with extra-renal
	898	Other - specify
	2000	Misc. bypass

© 2026 Yu ME et al. *JAMA Network Open*.

eFigure 1. Flow diagram of study cohort

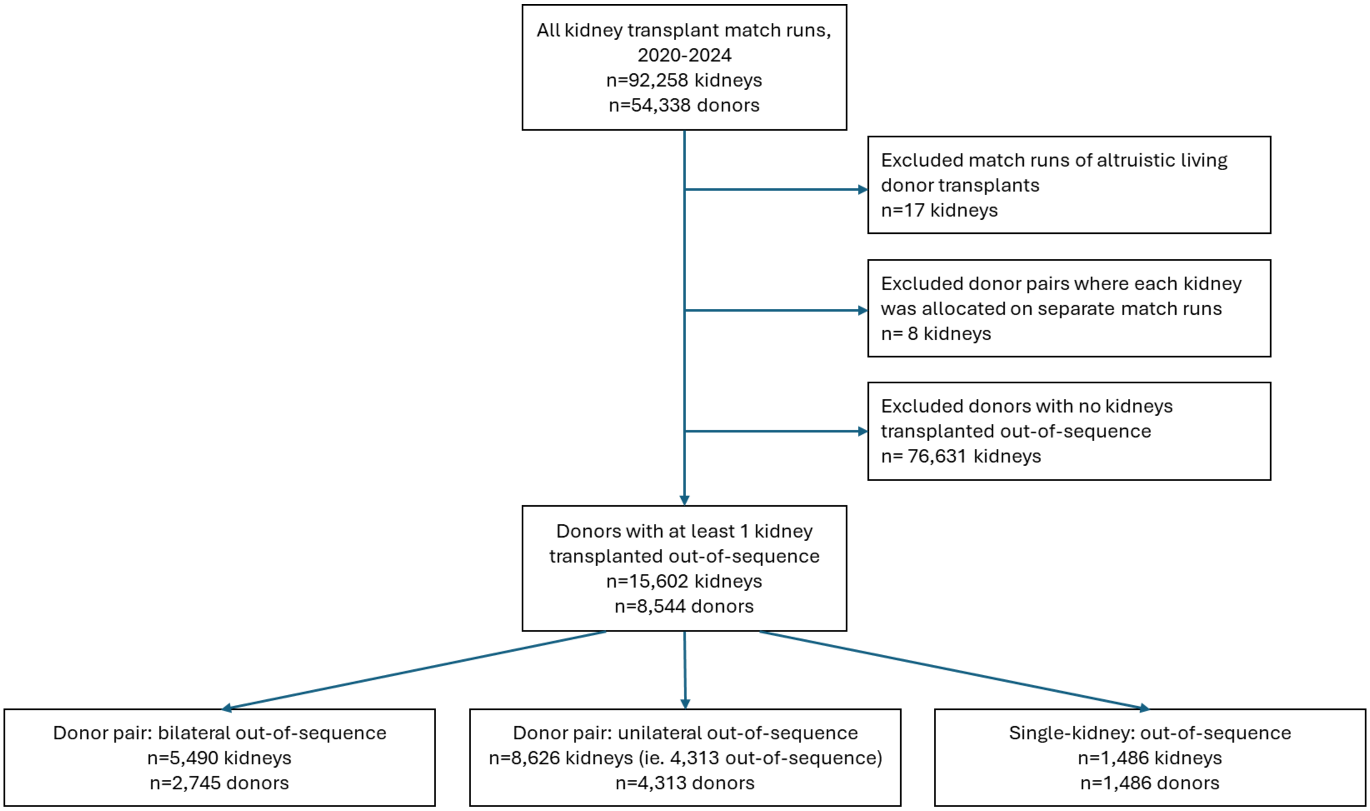

© 2026 Yu ME et al. *JAMA Network Open*.

eFigure 2. Distribution of the first appearance of an out-of-sequence refusal code (on a logarithmic scale) among all out-of-sequence transplants

Each boxplot represents an organ procurement organization (OPO) and the red marker indicates the median sequence number on a logarithmic scale.

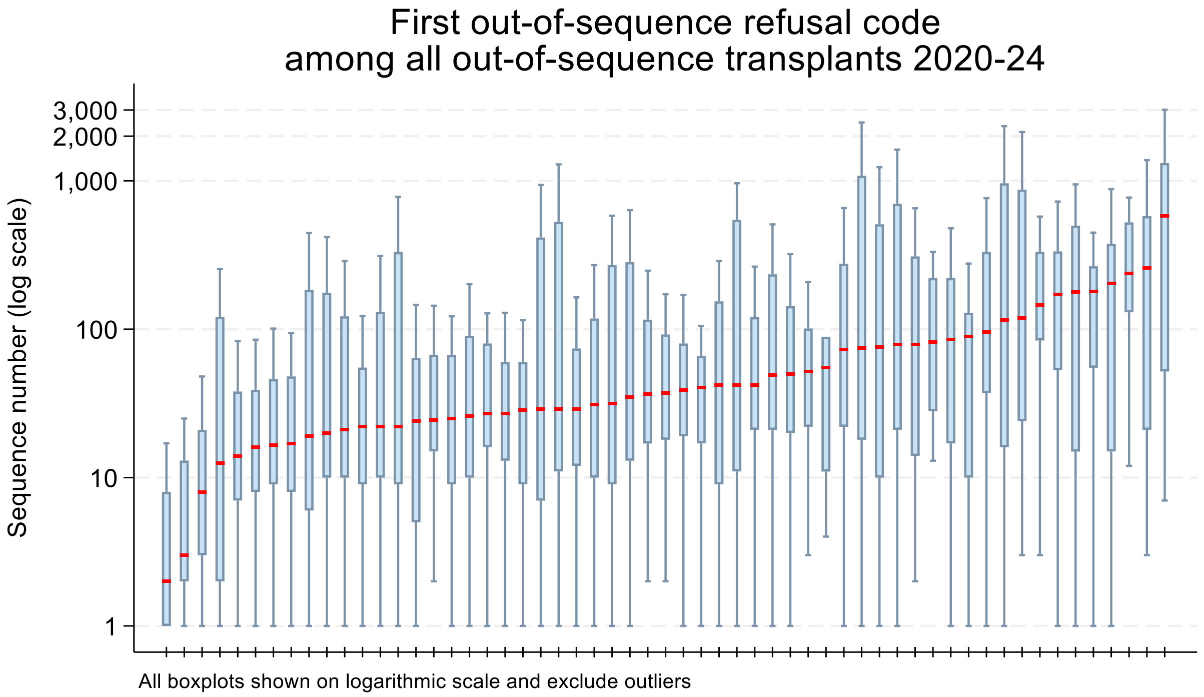

© 2026 Yu ME et al. *JAMA Network Open*.

eFigure 3. Annual proportion of refusal codes used before the first appearance of an out-of-sequence code among donor pairs with unilateral transplants

Transplants are categorized in ranges by the sequence number of their first out-of-sequence code. Refusal codes are categorized into recipient, donor history, biopsy findings, organ damage or anatomy, logistic, cold ischemia time, or other reasons (see Supplemental Table 1 for full list of refusal codes).

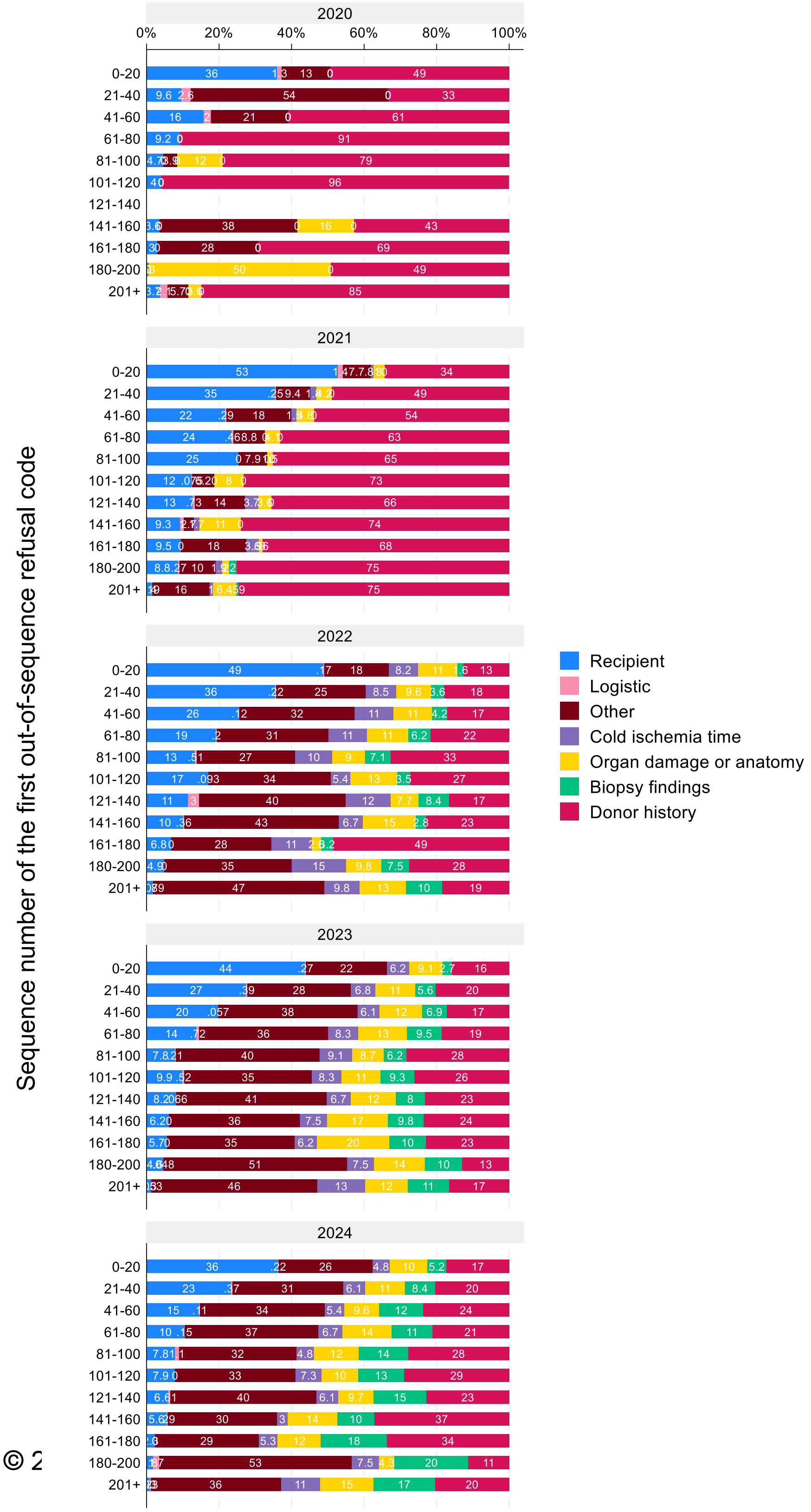

© 2

eFigure 4. Proportion of refusal codes used before the first appearance of an out-of-sequence code among donor pairs with unilateral transplants

Transplants are categorized in ranges by the sequence number of their first out-of-sequence code. Refusal codes are categorized into recipient, donor history, biopsy findings, organ damage or anatomy, logistic, cold ischemia time, or other reasons (see Supplemental Table 1 for full list of refusal codes).

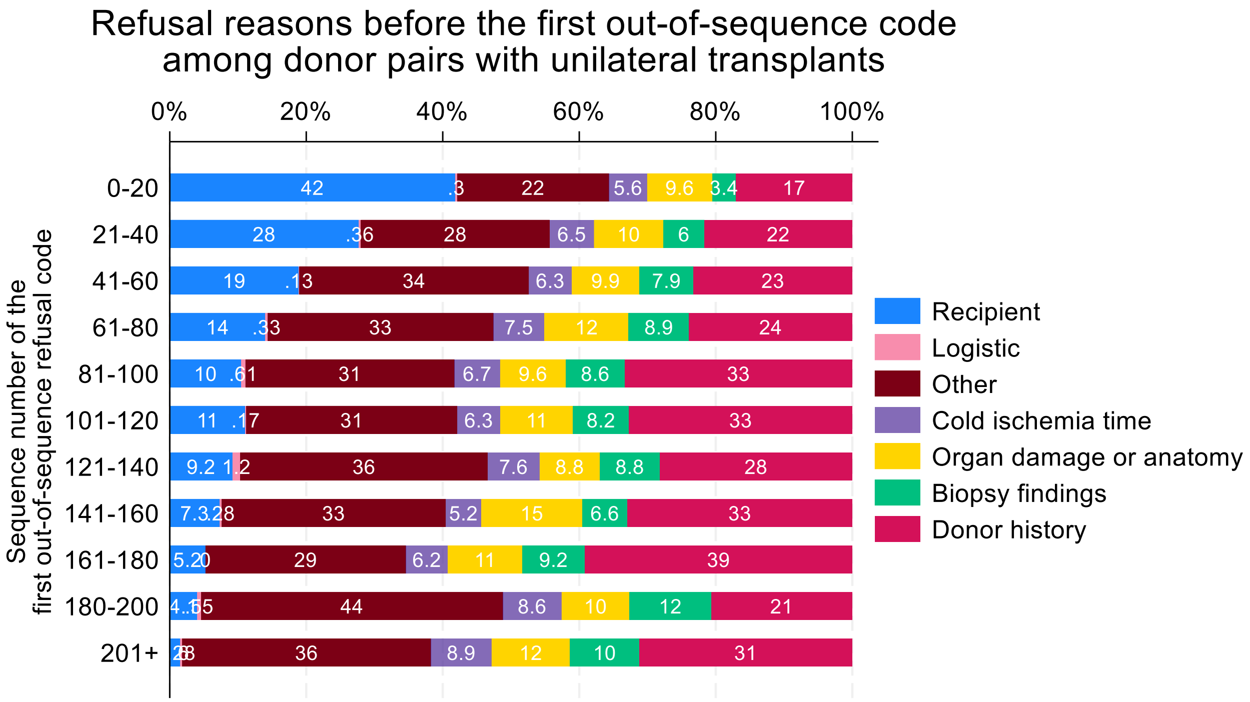

© 2026 Yu ME et al. *JAMA Network Open*.

eFigure 5. Unadjusted and adjusted patient and graft survival after transplant among donor kidney pairs with unilateral out-of-sequence transplants

Estimates are obtained from an unadjusted Cox model with robust sandwich estimators to account donor pairs.

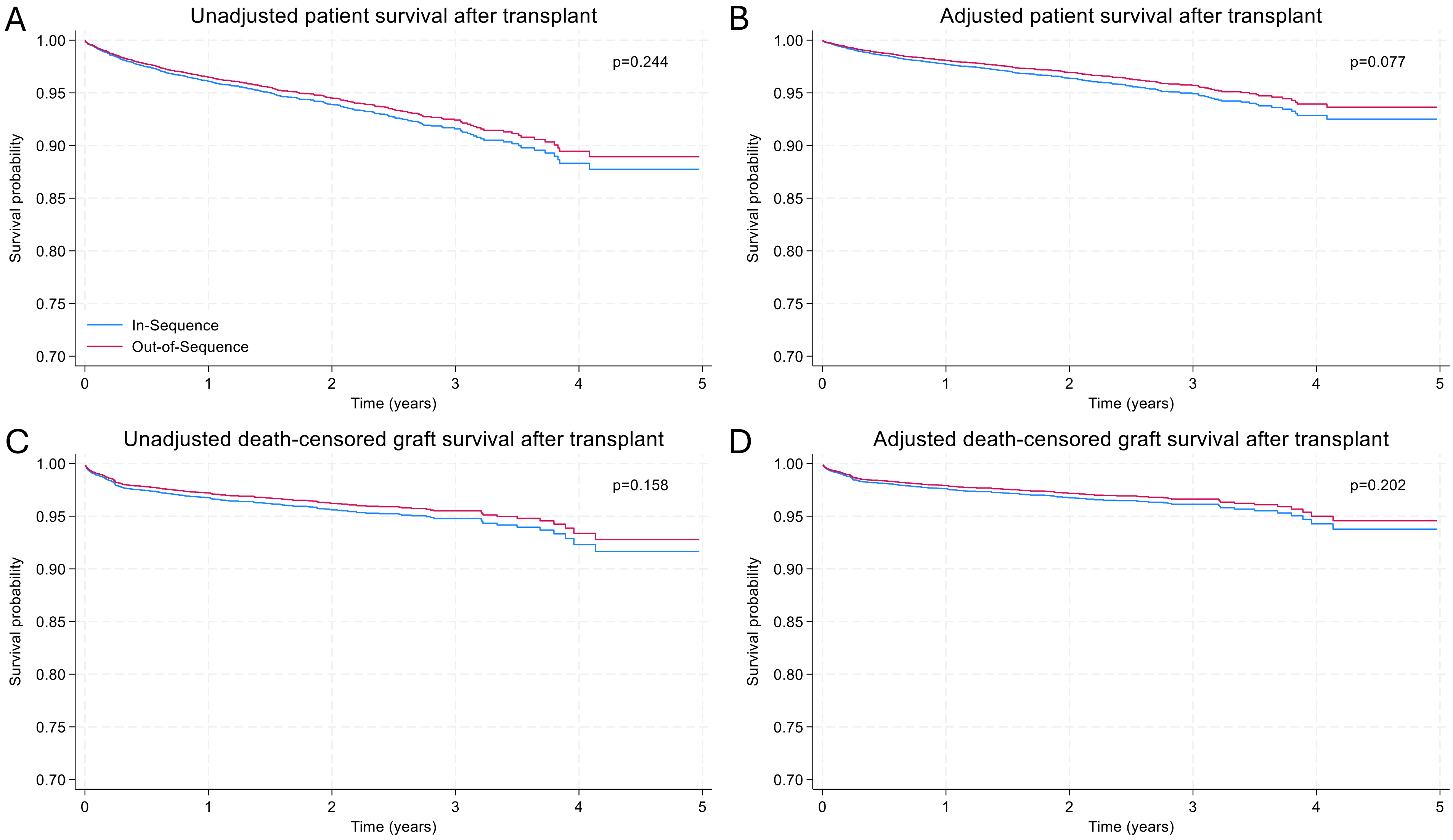

© 2026 Yu ME et al. *JAMA Network Open*.

eFigure 6. Unadjusted and adjusted patient and graft survival after transplant among donor kidney pairs with unilateral out-of-sequence transplants, stratified by Kidney Donor Profile Index (KDPI)

Estimates are obtained from an unadjusted Cox model with robust sandwich estimators to account donor pairs.

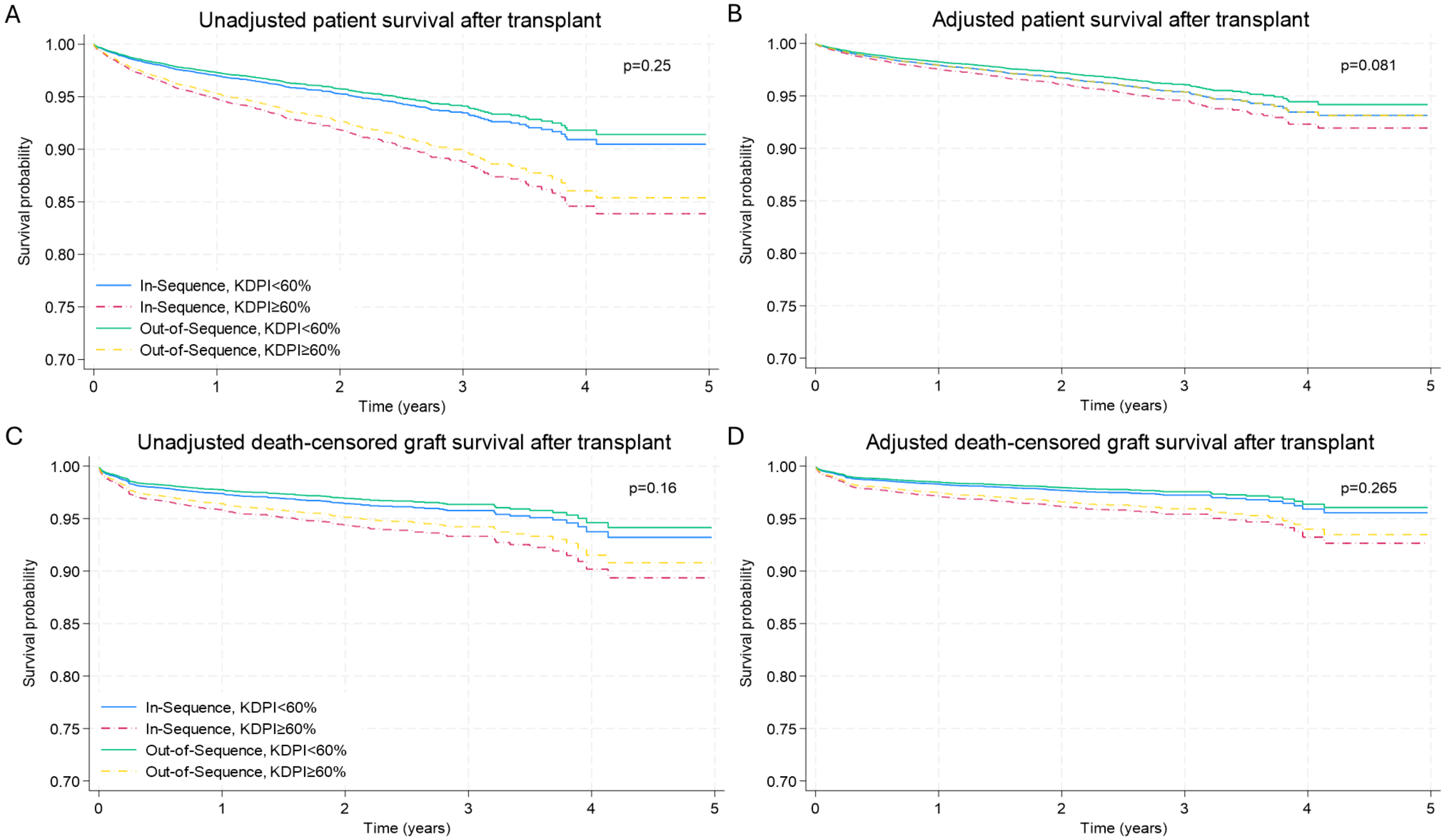

© 2026 Yu ME et al. *JAMA Network Open*.
