# Supplementary material for: Expedited Transplant Allocation Using a Paired Kidney Cohort
Source: JAMA Netw Open. 2026 Mar 4;9(3):e260257. doi: 10.1001/jamanetworkopen.2026.0257 (PMC12961519; doi:10.1001/jamanetworkopen.2026.0257)
Supplement: Supplement 2. — Data Sharing Statement [file jamanetwopen-e260257-s002.pdf]

Data Sharing Statement

Yu. Expedited Transplant Allocation Using a Paired Kidney Cohort. *JAMA Netw Open*. Published March 04, 2026. doi:10.1001/jamanetworkopen.2026.0257

Data
Data available: No

Additional Information
Explanation for why data not available: Data is available upon request and data use agreement with SRTR.
